# Current Hospital Topics

**Published:** 1906-10-13

**Authors:** 


### HOSPITAL ADMINISTRATION.

### CONSTRUCTION AND ECONOMICS.

### CURRENT HOSPITAL TOPICS.

**The Hospital Sunday Fund.**

In an interview with Sir Edmund Currie, published in the *Daily Mirror*, he is stated to have said that the falling off of from 15 to 30 per cent. in the amount contributed to the Hospital Sunday Fund in 1906, which represents a sum of nearly £20,000, is solely owing to the growing popularity of the week-end habit, which takes the people who can afford to give away from town. We should be inclined to say that there were other causes, including continued absence of business in the City of London and the diminished resources of Londoners generally, owing to the long depression which has prevailed and increased each year for several years past. There can, however, be no doubt that the week-end exodus does have a material influence on the amount of business done in London now and formerly, and an inspection of places of worship on Sundays will show that Sir Edmund Currie has certainly good ground for the opinion he has expressed. We are glad, therefore, to note that the energetic Secretary of the Hospital Sunday Fund has decided to make a special effort to reach the absentees, and if he succeeds we have no doubt that the result will be eminently satisfactory.

**The Hospitals and the Sunday and Saturday Funds.**

Once again the cry has been raised that "the funds subscribed to hospitals are diminishing owing to the Hospital Sunday and Saturday Funds, which have done serious damage to many well-deserving hospitals." This statement is made in the *Globe* by a gentleman, Mr. Gerard Van de Linde, who is a hospital auditor, and who might have used his knowledge of figures and his access to hospital books to prevent him from being the means of circulating an error which has been exposed and confuted over and over again. We have shown in these columns on more than one occasion that the revenues of the metropolitan hospitals have largely increased in the last ten years, a fact which in itself would seem to disprove the statement referred to. Proof becomes positive when the case of an individual hospital is taken, and the facts are placed beyond doubt by the production of actual figures. To prove his point the critic must show that before the institution of Hospital Sunday the total amount received by the particular hospital from congregational collections was greater than the average of the contributions now received each year from the funds. He must further prove that over a number of years the income of a particular hospital shows a material falling off in the total revenue derived from voluntary contributions; and, further, that the management of the hospital in question throughout the whole period has been good and efficient, and that sufficient energy has been thrown into the effort to raise money from voluntary sources. We shall be very glad to publish a statement signed by Mr. Gerard Van de Linde on the lines indicated in proof of the correctness of his statement, or to find room in our columns for a letter withdrawing that statement, should he find, on closer examination, as we are confident he must do, that the facts do not support the statement in question.

**Hospital Overbuilding.**

Many people hold the opinion, rightly or wrongly, that there has been an excess of expenditure upon hospital buildings in recent years which ought to have been avoided. The Glasgow *Evening News* reports a meeting of the Kilmacolm Parish Council, where a discussion arose as to the payment of the Council's proportion of the cost of the new Renfrewshire Combination Poorhouse and Hospital at Crookston. The contributing authorities appear to have agreed that each should take a certain number of shares, or billets, which the members understood to mean an undertaking to defray the cost of providing the number of beds which on an average they would require to meet the demands of the population for which they were responsible. Mr. John Lang, the Council's representative on the hospital committee, explained, however, that an extraordinary mistake had been made, for the space covered by buildings was exactly double that which was required. The consequence was that the Kilmacolm authority would have to pay for 14 beds instead of for seven, which were required, and that in the case of one parish the expense entailed by providing 80 beds would have to be supplied by the ratepayers, although only 20 beds were needed by the people in the district. Here surely is an opportunity for the Local Government Board to institute an inquiry and to surcharge the persons who may be found responsible for this wasteful and unnecessary expenditure upon buildings which are not wanted, and will probably never be filled by patients at all. The old joke that when attaining manhood a Scotsman goes south to despoil the Saxon would seem to have some measure of justification, for the practice seems to have unduly drained Scotland of the shrewd minds and thrifty men which formerly made the country famous in the history of the world.

**The Municipalisation of Hospitals.**

Miss Margaret McMillan has an article in the *Labour Leader* which indicates where the Socialists would lead us. Her immediate topic is the declaration of the trades unionists in favour of the children, which secures that a certain amount of medical inspection of children in the public schools is now to be compulsory. This inspection will not touch, in Miss McMillan's view, the great army of suffering children who cannot be sent to the hospitals, who are able to go to school, though these cases are tedious and require attention and skilled treatment for weeks. If such cases are sent to a hospital for free advice it excites the patient, and this excitement, coupled with the impatience of the doctor due to over-work, combine to secure that such cases are "not really treated in any true sense." The children hate the process, and the parents yield to their desire and keep them away from the hospital. Here we think Miss McMillan must be in error, seeing the ever-increasing number of cases which flock to the hospitals every day. Miss McMillan thinks that the medical inspection of children must prove a remedy, because it will result in the need for treatment of an army of school children becoming pressing. The municipality will then supply "a certain kind of hospital in connection with schools, manned in time by child specialists, and developing an order of talent that will be consecrated to the young." These municipal hospitals will be supported not by charity, but by the State. Miss McMillan declares that "the hospitals of to-day are the pride of England, and England has good cause to be proud of them; yet they break down at a certain point. They involve a waste of money, as well as of human life that can be avoided only when the care of the sick is the concern, not of the wealthy and philanthropic, but of the whole nation." We shall have something more to say on this subject, but we may here remark that if every public elementary and secondary school is to have a State hospital attached to it, and to give free treatment to every child attending such school, in addition to the free treatment which is now given by the voluntary hospitals, the nation will soon have to import practitioners, because our own native supplies must cease from inanition, due to starvation engendered by the impossibility of any doctor obtaining a living by his profession in these islands. The sentimental Socialist is becoming more dangerous to human life and human happiness than any dangerous explosive known to mankind.

